# The Innate Immune cGAS-STING-Pathway in Cardiovascular Diseases – A Mini Review

**DOI:** 10.3389/fcvm.2021.715903

**Published:** 2021-07-26

**Authors:** Lavinia Rech, Peter P. Rainer

**Affiliations:** ^1^Division of Cardiology, Department of Internal Medicine, Medical University of Graz, Graz, Austria; ^2^BioTechMed Graz, Graz, Austria

**Keywords:** STING, interferon, DAMPs, cardiovascular, inflammation, cGAS

## Abstract

Inflammation plays a central role in cardiovascular diseases (CVD). One pathway under investigation is the innate immune DNA sensor cyclic GMP-AMP synthase (cGAS) and its downstream receptor stimulator of interferon genes (STING). cGAS-STING upregulates type I interferons in response to pathogens. Recent studies show that also self-DNA may activate cGAS-STING, for instance, DNA released from nuclei or mitochondria during obesity or myocardial infarction. Here, we focus on emerging evidence describing the interaction of cGAS-STING with cardiovascular risk factors and disease. We also touch on translational therapeutic opportunities and potential further investigations.

## Introduction

Cardiovascular diseases (CVD) are one of the large health problems in our societies. The 2015 incidence for CVD in the European Society of Cardiology member countries was 11 million people with a prevalence of around 83.5 million (1). CVDs are the major global cause of death. Worldwide, more than 17 million people die from CVD each year (2, 3), 4 million of which in Europe (2). The relation between inflammatory and immune phenomena and the pathophysiology of CVD is receiving increasing attention recently. Its role in disease progression is evident in several conditions (3–5). For example, immune phenomena comprising both innate and adaptive responses are central in the development of atherosclerosis (6) and a landmark clinical trial demonstrated for the first time in 2017 that specifically targeting inflammation reduces cardiovascular events in high-risk subjects (7).

Inflammation and immune phenomena are also intricately involved in myocardial infarction healing and the progression of heart failure (8, 9). Early myocardial infarction remodeling is a wound healing response with the massive influx of myeloid cells from extra-cardiac reservoirs (10). These innate immune cells clear necrosis and pave the way for the establishment of a functional scar. Exuberant responses, e.g., after inhibiting regulatory cytokines such as TGF-β, are detrimental, and preclinical trials demonstrate the benefit of suppressing these (11, 12). However, clinical trials that sought to translate this to clinical use failed (13). Inflammatory processes are not only detrimental in injured tissues. In fact, they are often a requisite for repair and regeneration. The key is the right balance of proinflammatory and antiinflammatory responses in terms of magnitude and timing (12, 14). For instance, macrophages can be proinflammatory (M1 like) and required to clear damaged tissue, while pro-reparative M2 like macrophages mitigate inflammatory responses and are essential for ensuing healing processes (15, 16). In chronic heart failure, proinflammatory cytokines such as IL-1, IL-6, and TNFα are elevated and may contribute to disease progression (17), however, clinical studies targeting cytokines such as TGFα in heart failure patients failed in the past (18).

## Innate Immunity and the cGAS-Sting Pathway

The innate immune system is our first-line defense and consists of humoral and cellular parts. Cellular components are phagocytes like neutrophils, eosinophils, and macrophages, as well as natural killer cells and dendritic cells (19, 20). The humoral system includes the complement system and natural antibodies as well as cytokines like interferons (IFNs), interleukins (ILs), tumor necrosis factors (TNFs), and transforming growth factors (TGFs) (20–22). Triggers initiating inflammation are pathogen-associated molecular patterns (PAMPs) derived from infectious agents, or non-infectious damage-associated molecular patterns (DAMPs). These molecular patterns can consist of different components like cell wall components, proteoglycans, or nucleic acids. DNA e.g., can originate from extracellular sources such as viruses, bacteria, or dying cells. However, DNA can also be derived from intracellular sources such as damaged nuclei or mitochondria. What these sources have in common is that DNA is present in compartments where it is out-of-place. DNA sensors are, for instance, the Toll-like receptor 9 (TLR9), absent in melanoma 2 (AIM2), or interferon gamma-induced 16 (IFI16) (23). cGAS is another example for a DNA sensor.

**Figure 1:**
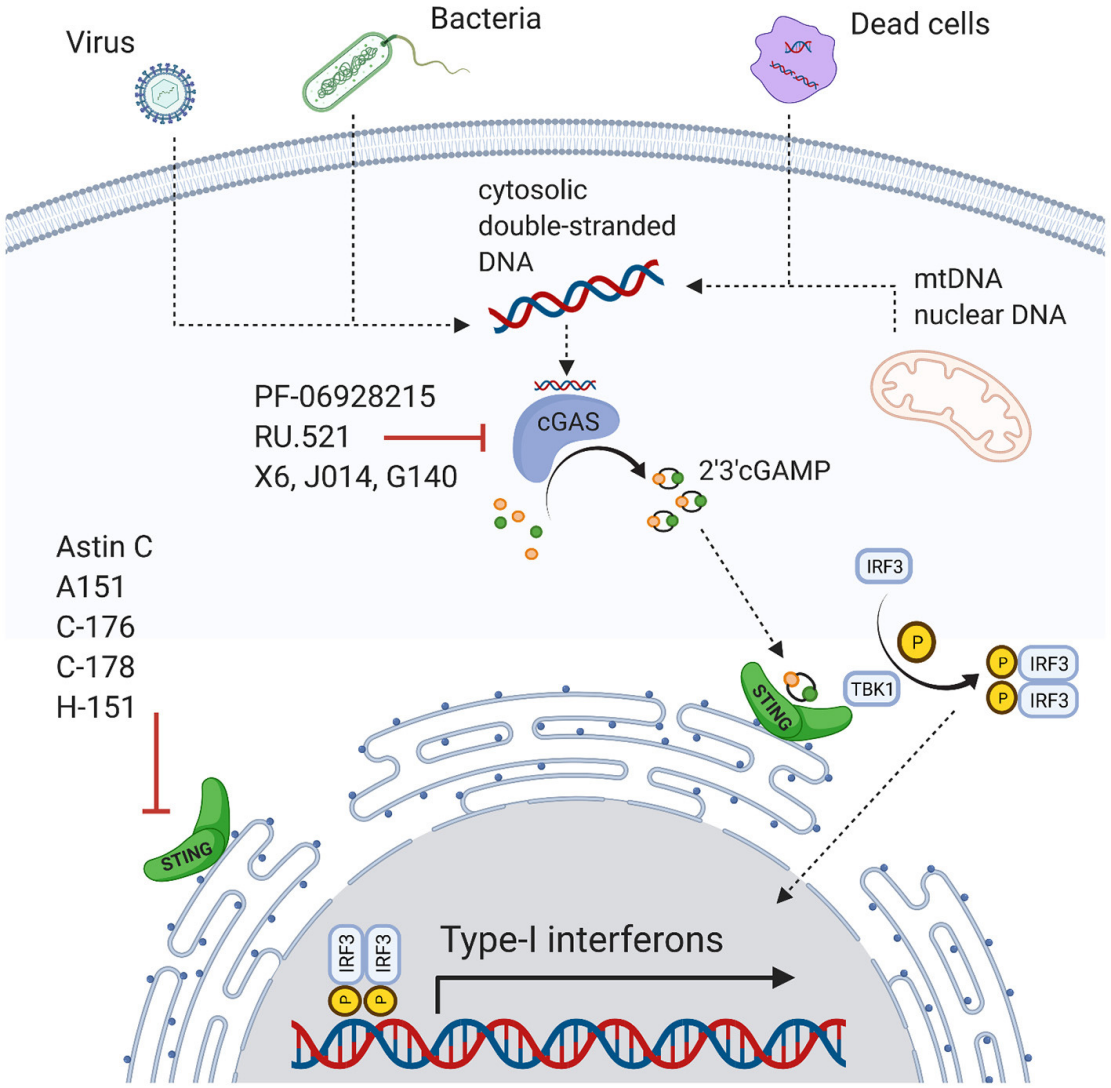
Overview of the cGAS-STING pathway and inhibitors.

cGAS is a 63kDa protein predominantly localized in the cytoplasm during the cell cycle's interphase (24). It was initially described as a defense mechanism against viral and bacterial infections by binding foreign DNA and transforming ATP and GTP to the second messenger cyclic-GMP-AMP (cGAMP) (Figure 1) (25–36). cGAMP activates the STING receptor (36–38). STING is a receptor protein with three isoforms ranging from 9–34 kDa and is localized at the endoplasmic reticulum (39). STING activates the TANK-binding kinase 1 (TBK1), which phosphorylates the transcription factor interferon releasing factor 3 (IRF3) (35–37). Ultimately, IRF3 induces the transcription of type I interferons (25, 37), which in turn activate several signaling cascades including activation of the IFN-α receptor 1 (IFNAR1) and the transcription of IFN-stimulated genes (ISGs) (40, 41).

Regulatory mechanisms are necessary to attenuate excessive proinflammatory stimulation. This is also true for the cGAS-STING pathway. For instance, cytosolic deoxyribonuclease degrades cytosolic DNA and ensures that minute amounts of free DNA do not trigger the full inflammatory cascade (42). Additionally, an intact cell's compartmentalization restricts nuclear or mitochondrial DNA sensing by cytosolic sensors (42). However, the regulatory capacity is limited. Genomic instability and nucleic damage can release DNA in considerable amounts into the cytosolic compartment activating cGAS (36, 38, 43). ER stress also actives STING and IRF3 (31, 44) and promotes autophagy in stressed cells, e.g., through direct interaction of cGAS with Beclin-1(31, 45). STING activated T-cells can further induce a type I IFN response eliciting apoptosis (46). Interestingly, even in T cell-derived cancer cells, this process is still functional and represents a therapeutic approach (47). Similarly, STING may induce apoptosis in malignant B cells (48). Activating the cGAS-STING pathway also improves the outcome of solid tumors, for instance in metastatic breast cancer, by enhancing the immune response against tumor cells (49, 50).

Another area where cGAS-STING is under investigation is infectious disease. For instance, virus infections like hepatitis B (51), Dengue (52), and HIV (31, 38) or bacterial infections like tuberculosis (53) and Streptococcus pyogenes infections (38).

An increased amount of self-DNA released into the cytosol by autoimmune diseases activates the cGAS-STING pathway as well, e.g., in Systemic Lupus Erythematosus or Aicardi-Goutières syndrome (31, 34, 43, 54, 55). Another autoinflammatory disease without an increased amount of self-DNA is the STING-associated vasculopathy with onset in infancy (SAVI) with gain-of-function mutations in the STING gene (56).

## Evidence for Involvement of the cGAS-Sting Pathway in Cardiovascular Risk Factors and Disease

**Figure 2:**
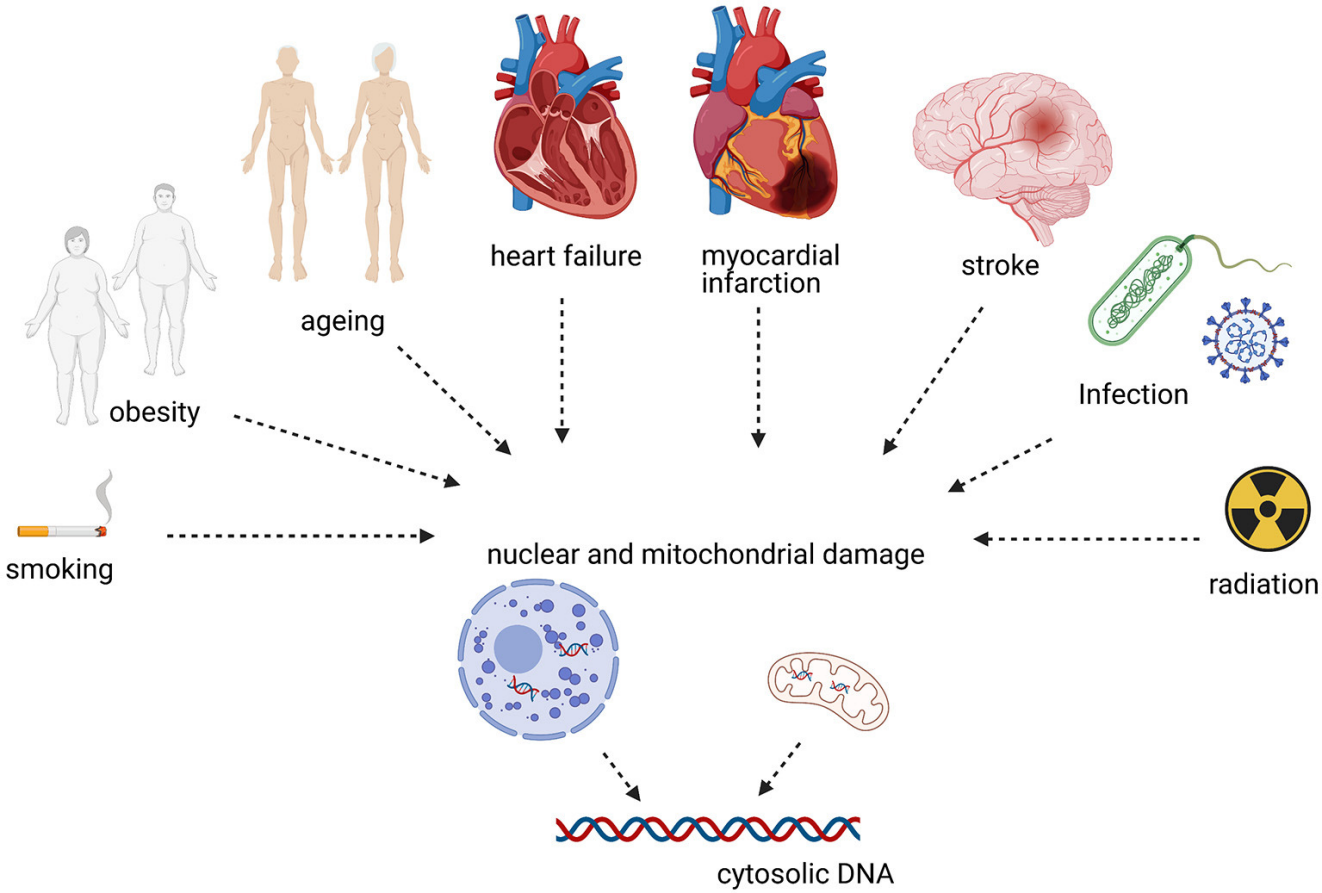
CVDs and risk factors associated with cytosolic DNA sensing.

Self-DNA can activate cGAS-STING in non-communicable, non-immune disorders such as CVD. Here, we give a concise overview of evidence linking CVD risk factors and disease to cGAS-STING (Figure 2).

### Risk Factors

#### Smoking

Liu et al. (57) showed that side-steam smoke exposure (SSE), a model for second-hand smoking (SHS), reduced fractional shortening (FS) in mice and increased left ventricular (LV) mass. Additionally, they investigated these effects on mice haploinsufficient for the autophagy protein Beclin 1 (Becn^+/−^). They found no difference between wild-type (WT) mice and Becn^+/−^ without SSE, but a significant reduction in FS and an increase in LV mass in Becn^+/−^ with SSE. On the cellular level, myocyte hypertrophy was present, myocardial TNFα and IL-1β increased, and cardiomyocyte peak shortening was reduced. This was associated with an increase of cGAS and STING protein expression, suggesting that this pathway is involved in the inflammatory process of SHS in WT and Becn^+/−^ mice and that this is exacerbated with impaired autophagy. Furthermore, the authors tested the cGAS inhibitor (PF-06928215) and STING inhibitor (Astin C) in their study. In WT mice with SSE, the inhibitors improved peak shortening significantly, while this effect was lost in Becn^+/−^ mice (57). Chronic ozone exposure, which mimics smoke-induced chronic obstructive pulmonary disease (COPD) and induces reactive oxygen species (ROS) and mitochondrial damage, may also be associated with the cGAS-STING signaling in humans (58).

#### Obesity

Another major risk factor for CVDs is obesity. Obesity is associated with endothelial inflammation (59) and induces proinflammatory responses in M1 macrophages, e.g., through elevated levels of palmitic acid (PA) in the blood (60). Mao et al. (61) investigated the influence of PA on cardiovascular endothelia and STING's role in this interaction. *In-vitro* experiments in human aortic endothelial cells demonstrate PA-induced mitochondrial damage and release of mitochondrial DNA (mtDNA) into the cytosol leading to cGAS STING pathway activation and IFN production. Silencing STING or IRF responses *via* small interfering RNA (siRNA) attenuates this response. These results were reiterated *in-vivo* as well. Wild-type mice on a high-fat diet (HFD) had a significant increase of IRF3 in adipose tissue and the aortic wall, which was reduced in STING-deficient (STING^gt/gt^) mice (61).

A recent study from Gong et al. supports cGAS-STING's effect on HFD associated cardiovascular dysfunction. They showed that WT mice with HFD had significantly reduced FS *in-vivo*, peak shorting in isolated cardiomyocytes, and cardiomyocyte hypertrophy. This was accompanied by elevation of TNFα, IL-1β, STING, and cGAS. Deletion of *Akt2* and *Ampk*α*2* (double knock-out, DKO), decreased phosphorylation of Unc-51 like autophagy activation kinase (ULK1) (62), which phosphorylates Beclin1 and thereby induces autophagy (63). Furthermore, cGAS/STING activation on HFD was amplified in DKO (62).

#### Aging

Inflammageing describes low-grade, chronic, and sterile inflammation that occurs with aging and is associated with CVDs (64). One trigger for this inflammatory process is the degeneration of DNA during aging.

Quan et al. (65) demonstrated that cGAS-STING regulates the senescence-associated secretory phenotype (SASP). SASP in aged hearts is primed by an increase in proinflammatory cytokines like IL-1β, IL-6, and IL-8, and release of mtDNA into the cytosol may induce SASP *via* cGAS-STING. Circulating mtDNA associated with age increases inflammatory SASP in aged hearts (66, 67).

Interestingly, patients suffering from the accelerated aging disease Hutchinson Gilford Progeria Syndrome (HGPS) often die from CVDs like myocardial infarction (MI) or stroke (68) and HGPS is associated with amplified interferon responses potentially *via* the cGAS-STING pathway (69–71). However, mutations can also be protective. The single nucleotide polymorphism (SNP) R293Q of the *STING* gene is protective in obesity-associated CVDs and other age-related diseases (72, 73).

### Established CVD

#### Heart Failure

Heart Failure (HF) is a clinical syndrome with symptoms and structural and/or functional cardiac abnormalities (68). It represents end stage disease in many CVDs like ischemia or hypertension.

In a model of non-ischemic pressure-overload induced heart failure (transverse aortic constriction, TAC) exhibiting hypertrophy, cardiac dysfunction, and fibrosis expression of STING, IFNα and IFNβ were increased (74). In STING knock-out (STING-KO) mice, levels returned to baseline levels (74). Neonatal rat cardiomyocytes treated with angiotensin II had increased levels of STING, IFNα, and IFNβ. STING inhibition *via* siRNA resulted in a significant reduction of IL-6, IL-1β, TNFα, IFNα, and IFNβ in these cells. Increased levels of STING, IFNα, and IFNβ were also seen in human samples of dilative and hypertrophic cardiomyopathies (74).

Another study confirmed these findings: the expression levels of cGAS, STING, IFN, and the IFN induced chemokines CXCL10, IFIT3, and ISG15 were significantly increased 3 days after TAC (75). Silencing cGAS *via* adeno-associated virus 9 (AAV9) resulted in a significant decrease of LV remodeling and fibrosis (75).

#### Myocardial Infarction

Two independent groups investigated the relevance of cGAS-STING in myocardial infarction healing. They demonstrate increased IFNβ1 expression and IRF3 phosphorylation and an increase in the expression levels of CXCL10, IRF7, STING, and cGAS after myocardial infarction (76, 77). This was attenuated by using knockout models for pathway members such as cGAS, STING, or IRF3. Interestingly, cGAS knock-out (cGAS^−/−^) did not reduce the universal proinflammatory cytokines IL-1β, TNFα, and IL-6 (76).

By using fluorescence reporter tagged cells, parabiosis experiments, and scRNAseq King et al. (77) demonstrated that cardiomyocyte cell death after MI leads to recruitment of interferon-inducible cells (IFNICs) with increased expression of IRF3-dependent genes from the blood to the heart and they identify these IFNICs as monocyte-derived cardiac macrophages that phagocytose cell debris. Disruption of pathway activation *via* genetic or pharmacologic means improves outcomes.

Cao et al. (76) treated WT and cGAS^−/−^ human macrophages with IFN stimulatory DNA. As expected, cGAS^−/−^ macrophages produced no cGAS and also no CXCL10 (76). CXCL10 was expressed in WT macrophages and associated with M1-like polarization. In contrast, M2 marker expression like CD163, IL-10, and CCL17 was increased in cGAS^−/−^ animals (76).

Both groups observed improved outcomes in cGAS^−/−^ mice compared to WT in terms of LV function and survival (76, 77), although, the survival benefit was more pronounced in INFAR and IRF3 knockout animals. IFNAR neutralization *via* antibodies mirrored survival and functional benefit.

Interestingly, Cao et al. showed increased myofibroblast activation and collagen deposition in cGAS^−/−^ mice after MI and propose this enhances functional scar generation.

Cao et al. also provide data demonstrating high myocardial levels of cGAS and CXCL10 in human end stage ischemic heart failure patients that are decreased back to near normal levels by unloading the left ventricle by means of mechanical circulatory support *via* left ventricular assist devices (LVADs).

#### Stroke

Li et al. (78) showed that the cGAS-STING pathway is also involved in stroke in an *in-vivo* model with middle cerebral artery occlusion (MCAO). They observed increased levels of cGAS and STING in the infarcted brain area. Using a small synthetic oligodeoxynucleotide, A151 (TTAGGG), which inhibits cGAS, this was reduced to the levels of sham-operated mice. Additionally, A151 reduced IL-1β levels, reduced infarct size and improved cognitive function (78).

#### Cardiovascular and Systemic Infection

Li et al. (79) described upregulation of STING and phosphorylated IRF3 in an *in vitro* model of sepsis induced cardiomyopathy (SIC) using neonatal rat cardiomyocytes. Treating cells with siRNA against STING resulted in a decrease in IRF3 phosphorylation. In an *in vivo* model of SIC using LPS injection STING-KO reduced CK-MB, IL-1β, and TNFα levels and improved EF, FS, and survival. Likewise, other investigators found that the small cGAS inhibitor molecule RU.521 improved LPS induced SIC (IRF3 phosphorylation, IL-1β, IL-6, TNFα expression, apoptosis, left ventricular function, and survival) (80). Lastly, selenium supplementation appeared to ameliorate LPS-induced SIC *via* STING (81).

In Chagas cardiomyopathy, Choudhuri et al. showed that extracellular vesicles from Trypanosoma cruzi infected cells lead to increased levels of IL-1β, IL-6, and TNFα in macrophages. Using different inhibitors, including the cGAS inhibitor PF-06928215, they detected a significant decrease in the levels of IL-1β, IL-6c, and TNFα (82).

Further, there is speculation that COVID-19 infection may lead to prolonged cGAS-STING pathway activation in leucocytes (83) and increased leucocyte infiltration was present in the majority COVID-19 patient's hearts in an autopsy studie (84).

#### Radiation Injury

Radiation produces DNA damage, which can be sensed in the cytosol by cGAS (85). Phillipp et al. (86) studied the effect of radiation on cultured human coronary artery endothelial cells. With increasing radiation up to 10 Gy, the expression levels of STING and ISG15 increased continuously after 1 week as well as ISG15 and cGAS up to a dose of 2 Gy. This may have clinical implications as radiation therapy for breast cancer may result in up to 20 Gy delivered to the left anterior descending coronary artery (LAD) (87).

## Conclusion and Outlook

cGAS-STING is involved in the pathophysiology of cardiovascular disease and risk factors. This ranges from conditions with cell death and massive release of DAMPs such as myocardial infarction or stroke to chronic conditions where inflammatory responses are mildly increased over longer periods such as heart failure. This may have translational implications, as pharmacologic agents are available and have been tested for non-cardiovascular diseases. Inhibitors of cGAS include PF-06928215, A151, RU.521, J014, G140, or X6 (80, 88–90). Direct STING inhibition also seems promising (91) and antagonists include Astin C, C-176, C178, and H-151 (88, 92, 93). However, potential adverse effects need to be studied. As cGAS and STING agonists are used for cancer and viral infection treatments (48–51), inhibition may promote these conditions. Furthermore, pathways are more complex and promiscuous than mentioned here, and inhibitors targeting other molecules may impact cGAS-STING too, for instance the ALK inhibitor LDK378 (94).

In conclusion, CVD and risk factors modulate cGAS-STING and this may contribute to disease progression. Targeting pathway members may be useful to attenuate excessive inflammation, e.g., ischemic injury to the heart or brain.

## Author Contributions

LR and PR wrote the manuscript. All authors contributed to the article and approved the submitted version.

## Conflict of Interest

The authors declare that the research was conducted in the absence of any commercial or financial relationships that could be construed as a potential conflict of interest.

## Publisher's Note

All claims expressed in this article are solely those of the authors and do not necessarily represent those of their affiliated organizations, or those of the publisher, the editors and the reviewers. Any product that may be evaluated in this article, or claim that may be made by its manufacturer, is not guaranteed or endorsed by the publisher.
